# Biotransformation and chemotaxis of 4-chloro-2-nitrophenol by *Pseudomonas* sp. JHN

**DOI:** 10.1186/s12934-014-0110-7

**Published:** 2014-08-12

**Authors:** Pankaj Kumar Arora, Hanhong Bae

**Affiliations:** School of Biotechnology, Yeungnam University, Gyeongsan, Gyeongbuk, 712-749 Republic of Korea

**Keywords:** 5-Chloro-2-methylbenzoxazole, Biotransformation, Chemotaxis, 4-Chloro-2-nitrophenol, *Pseudomonas* sp.

## Abstract

*Pseudomonas* sp*.* JHN decolourized and biotransformed 4-chloro-2-nitrophenol (4C2NP) in the presence of additional carbon source. The effect of the various concentrations of the 4C2NP was studied on the decolourization of 4C2NP by *Pseudomonas* sp. JHN. It was observed that strain JHN decolourized and biotransformed 4C2NP up to concentration of 0.6 mM. Gas chromatography and gas chromatography-mass spectrometry detected 5-chloro-2-methylbenzoxazole as a major metabolite of the co-metabolism of 4C2NP. Furthermore, strain JHN exhibits positive chemotaxis toward 4C2NP based on the drop plate and capillary assays. This is the first report of the chemotaxis toward 4C2NP by any bacterium.

## Introduction

4-Chloro-2-nitrophenol (4C2NP) is an anthropogenic toxic compound that has been detected in various contaminated sites over the world [1]. Several physicochemical methods including advanced oxidation processes have been used to decontaminate wastewater containing 4C2NP [2]. These physicochemical methods are not so much effective as compare to bacterial bioremediation which has been identified as an emerging approach for degradation of various xenobiotic compounds [2].

Despite the fact that 4C2NP is a recalcitrant molecule to bacterial attack due to presence of two electron withdrawing groups, a few reports have been published dealing with the bacterial degradation of 4C2NP [3–6]. Arora *et al*. [1] studied complete mineralization of the 4C2NP by *Exiguobacterium* sp. PMA that degraded it with stoichiometric release of chloride and ammonium ions. The degradation pathway involves the reduction of the 4C2NP into 4-chloro-2-aminophenol that dehalogenated to aminophenol which was degraded further with release of ammonia. A genetically engineered bacterium, *Pseudomonas* sp. N31 mineralized 4C2NP with release of chloride and nitrite ions and formation of 4-chlorocatechol [3]. The biotransformation and detoxification of 4C2NP has been studied in two *Bacillus* species isolated from two different ecological niches [4,5]. A marine bacterium, *Bacillus* sp. MW-1 and a soil bacterium, *Bacillus subtilis* RKJ 700 decolourized 4C2NP in the presence of additional carbon source. Both of the strains biotransformed 4C2NP into 5-chloro-2-methylbenzoxazole via formation of 4-chloro-2-aminophenol and 4-chloro-2-acetaminophenol [4,5]. 4-Chloro-2-aminophenol and 4-chloro-2-acetaminophenol were also identified as the metabolites in the degradation pathway of 4C2NP using the co-culture of two bacteria, *Enterobacter cloacae* and *Alcaligenes* sp. TK-2 [6]. In this communication, we describe the biotransformation and chemotaxis of 4C2NP by a previously isolated bacterium, *Pseudomonas* sp. JHN.

## Materials and methods

### Chemicals

4C2NP was purchased from Aldrich (Milwaukee, Wis.). 5-chloro-2-methylbenzoxazole was purchased from Across Organics. All other chemicals were used of high purity grade.

### Bacteria and growth conditions

Bacteria used in this study was *Pseudomonas* sp. JHN previously isolated from waste water collected from a chemically-contaminated area, India by an enrichment method [7]. This bacterium utilized 4-chloro-3-nitrophenol as the sole carbon and energy source [7]. However, this strain was unable to utilize 4C2NP as the sole source of carbon and energy but decolourized and transformed 4C2NP in the presence of additional carbon source. In this communication, we have monitored the ability of strain JHN to decolourize and bio-transformed 4C2NP in the presence of additional carbon source (i.e., glucose). This strain was grown on minimal media containing 4C2NP and glucose under shaking conditions (200 rpm) at 30°C. The composition of minimal media was exactly same as described previously [4,5,7].

### Growth and decolourization studies

To monitor the effects of different concentrations of 4C2NP on the growth of strain JHN and decolourization of 4C2NP, strain JHN was grown on 500 ml Erlenmeyer flask containing 200 ml minimal media, 10 mM glucose and appropriate concentration of 4C2NP (0.2 mM/0.4 mM/0.6 mM/ 0.8 mM). Samples were collected at regular intervals and the bacteria growth was monitored by taking absorbance at 600 nm [4,5].

For decolourization studies, the samples collected at regular intervals were centrifuged. The decolourization was monitored by the measuring the absorbance of supernatant at 420 nm using U.V-Visible spectrophotometer [5]. The percentage decolourization was calculated according to the formula as described previously [5]:

$$ \%\ \mathrm{Decolourization} = \left(\mathrm{Initial}\ \mathrm{Absorbance}\ \hbox{-} \mathrm{Absorbance}\ \mathrm{after}\ \mathrm{t}\mathrm{ime}\ \mathrm{t}\right) \times 100/\mathrm{Initial}\ \mathrm{Absorbance}. $$

### Identification of metabolites

Strain JHN was grown in 500 ml Erlenmeyer flask containing 200 ml minimal media, 0.6 mM 4C2NP and 10 mM glucose. Samples (50 ml) were collected at regular intervals (0, 12 and 16 h) and centrifuged at 8000 g for 15 min. The samples were extracted with ethyl acetate and analyzed by the gas-chromatography and the gas chromatography-mass spectrometry (GC-MS) by methods as described previously [7].

### Detection of ammonia, nitrite and chloride

The ammonium, nitrite and chloride ions were analyzed by the methods as described previously [4,5,7–9].

### Chemotaxis towards 4C2NP

The chemotactic response of *Pseudomonas* sp. JHN toward 4C2NP was investigated qualitatively with drop plate assay and quantitatively with capillary assay as described by Pandey *et al*. [10]. In drop plate assay, bacterial cells were grown in minimal media containing 0.6 mM 4C2NP and 10 mM glucose. The cells were harvested at mid-log phase (OD600 ~ 0.35) by centrifugation at 10000 rpm for 15 min. Harvested cells were washed twice with phosphate buffered saline, resuspended in minimal medium containing 0.3% bacto agar and poured into 96 mm petri plate [10]. Few crystals of 4C2NP were placed in the center of petri-plate and petri-plate was incubated at 30°C [10]. The chemotactic response was observed after 6 h of incubation. A positive response was indicated by the formation of concentric chemotaxis rings, due to bacterial cell accumulation encircling the crystals [7,10]. Capillary assay was performed as described previously [7]. The optimum concentration of 4C2NP for capillary assay was determined by performing assays at various 4C2NP concentrations (from 50-550 μM in 50 μM increments). In capillary assay, a 10 μl glass capillary was filled with a solution of desired concentration of 4C2NP (in chemotaxis buffer consisting of 100 mM potassium phosphate (pH 7.0) and 20 μM EDTA) and then inserted into a glass slide containing a suspension (10^13^cells/ml) of cells of strain JHN, and incubated at 30°C for 30 min [7,10]. The contents of the capillary tubes were then serially diluted and plated onto nutrient agar. Colony forming units (CFUs) were counted after 48 h incubation at 30°C. The strength chemotactic response was expressed in terms of the chemotaxis index, which is the ratio of the number of CFUs produced from the capillary containing the 4C2NP to CFUs produced from a control capillary (i.e. chemotaxis buffer without any chemotactic compound) [7,10]. Aspartate was used as the positive control.

## Results and discussion

**Figure 1:**
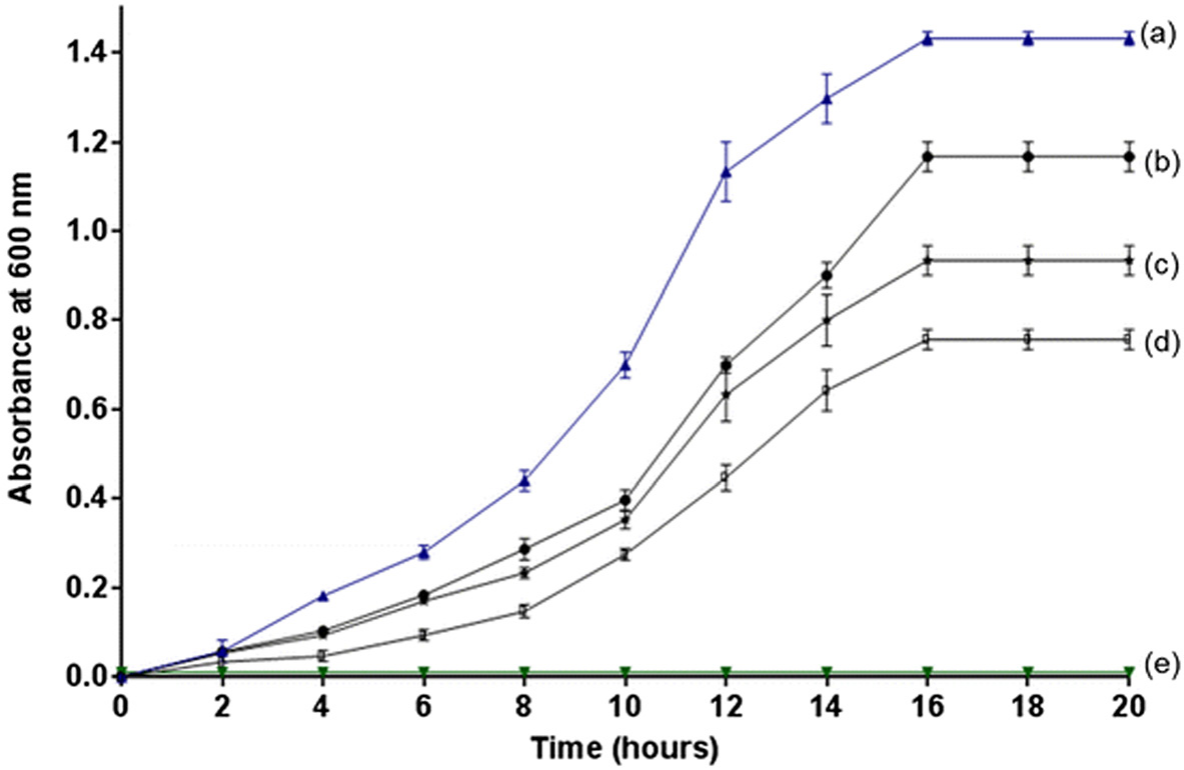
**Growth curves of** ***Pseudomonas*** **sp** ***.*** **JHN in different conditions.** Growth of *Pseudomonas* sp*.* JHN in **(a)** 10 mM glucose (as positive control), **(b)** 10 mM glucose and 0.2 mM 4C2NP, **(c)** 10 mM glucose and 0.4 mM 4C2NP, **(d)** 10 mM glucose and 0.6 mM 4C2NP and **(e)** 10 mM glucose and 0.8 mM 4C2NP.

A 4-chloro-3-nitrophenol-mineralizaing bacterium, *Pseudomonas* sp. JHN previously isolated from waste water collected from a chemically-contaminated area, India decolourized and biotransformed a yellow-orange coloured compound 4C2NP in the presence of glucose as the additional carbon source. We have monitored the effect of different concentrations of 4C2NP on the growth of *Pseudomonas* sp. JHN. Strain JHN was able to grow on 4C2NP up to concentration of 0.6 mM in the presence of 10 mM glucose. The growth of the cells of strain JHN was higher at the lower concentrations of 4C2NP as compared to higher concentrations (Figure 1). No bacterial growth was observed when the concentration of 4C2NP was 0.8 mM. This data suggests that the high concentrations of 4C2NP are toxic to the cells of strain JHN and inhibits the bacterial growth.

**Figure 2:**
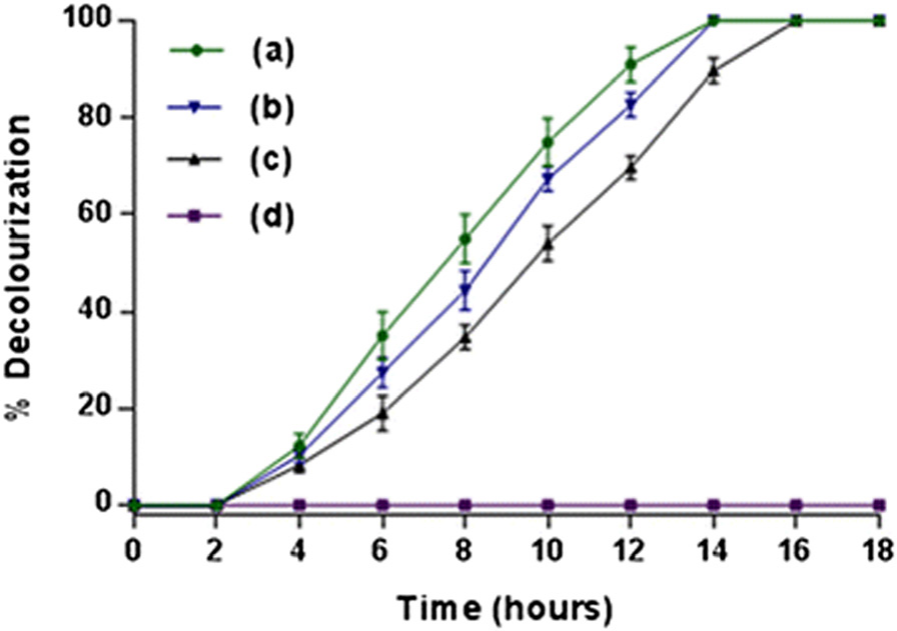
**Decolourization of 4C2NP by** ***Pseudomonas*** **sp. JHN at various concentrations.** Decolourization of the minimal media containing 10 mM glucose and **(a)** 0.2 mM 4C2NP, **(b)** 0.4 mM 4C2NP, **(c)** 0.6 mM 4C2NP, **(d)** 0.8 mM 4C2NP.

We have also investigated the effect of different concentrations of 4C2NP on decolourization by *Pseuodomonas* sp. JHN. Strain JHN decolourized 4C2NP up to a concentration of 0.6 mM. There was no decolourization at concentration above 0.6 mM due to the inhibition of the growth of the cells of strain JHN. Strain JHN decolourized 0.6 mM 4C2NP completely within 16 hours (Figure 2).

**Figure 3:**
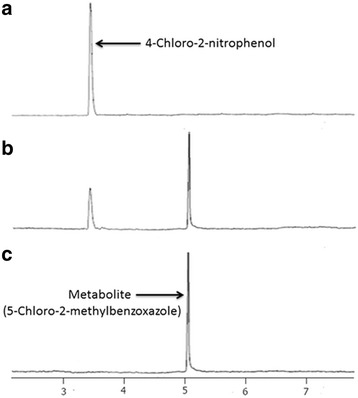
**Gas chromatography profiles of the samples collected at 0 h (a), 12 h (b) and 16 h (c) during the transformation of 4C2NP into 5-chloro-2-methylbenzoxazole by** ***Pseudomonas*** **sp** ***.*** **JHN.**

**Figure 4:**
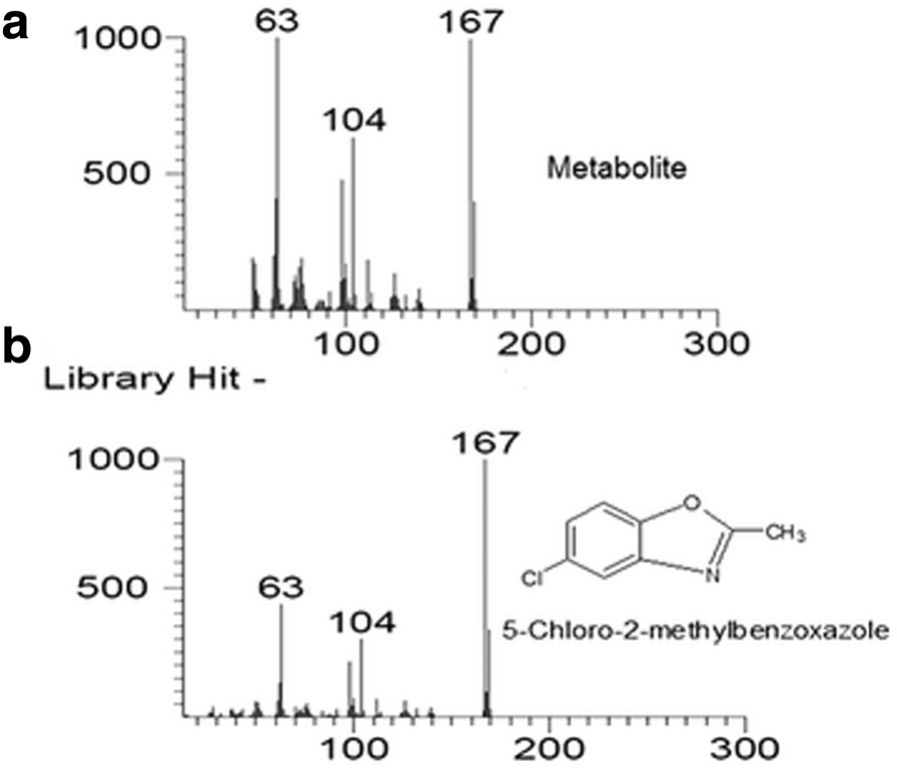
**NIST mass spectral library match of the GC-MS chromatogram of metabolite (a) with 5-chloro-2-methylbenzoxazole (b).**

**Figure 5:**
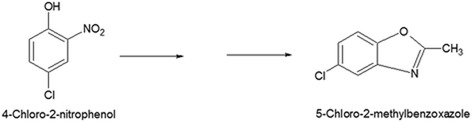
**A graphic representation of transformation of 4-chloro-2-nitrophenol into 5-chloro-2-methylbenzoxazole.**

GC results confirmed the complete depletion of 4C2NP with appearance of single metabolite (Figure 3). In the sample of the 0 h, only 4C2NP was detected. In the sample of 12 h, one peak of metabolite was appeared with a peak of parent compound. In the 16 h, the peak of 4C2NP was completely disappeared whereas the peak of metabolite was present. This data suggests that 4C2NP was completely transformed to metabolite. To identify the metabolite, GC-MS was carried out. The mass fragment of the metabolite that was observed at 167 m/z was subjected to the library search. NIST mass spectral library match showed that mass fragment of metabolite was exactly matched with to that of the 5-chloro-2-methylbenzoxazole (Figure 4). On the basis of the GC-MS, the transformation product was identified as 5-chloro-2-methylbenzoxazole. Previous studies have also been showed the formation of 5-chloro-2-methylbenzoxazole from 4C2NP by two *Bacillus* species [4,5]. The mechanism of formation of 5-chloro-2-methylbenzoxazole has also been studied in *Bacillus* sp. MW-1 and *Bacillus subtilis* RKJ 700. Both of the *Bacillus* spp. initially reduced to 4C2NP into 4-chloro-2-aminophenol that was further acetylated to 4-chloro-2-acetaminophenol [4,5]. 4-Chloro-2-acetaminophenol produced 5-chloro-2-methylbenzoxazole after cyclation. In the case of *Pseudomonas* sp. JHN, we could not detected 4-chloro-2-aminophenol and 4-chloro-2-acetaminophenol as biotransformation product due to the rapid transformation of 4C2NP to 5-chloro-2-methylbenzoxazole (Figure 5).

Several bacteria degraded 4C2NP via different mechanisms without formation of 5-chloro-2-methylbenzoxazole [1,3,6]. *Exiguobacterium* sp. PMA initially reduced 4C2NP to 4-chloro-2-aminophenol that further converted to 2-aminophenol which was further degraded via ring cleavage [1]. A genetically engineered bacterium, *Pseudomonas* sp. N31 degraded 4C2NP via the formation of 4-chlorocatechol [3]. The combination of *Enterobacter cloacae* and *Alcaligenes* sp. TK-2 degraded 4C2NP via formation of 4-chloro-2-aminophenol and 4-chloro-2-acetaminophenol [6]. In case of *Pseudomonas* sp. JHN, 5-chloro-2-methylbenzoxazole was formed which is more complex compound than 4C2NP. Further degradation of 5-chloro-2-methylbenzoxazole seems to be a challenging step due to its complex structure.

Several bacteria released chloride, nitrite and ammonium ions due to degradation of 4C2NP [1,3,6]. A genetically engineered bacterium, *Pseudomonas* sp. N31 mineralized 4C2NP with release of chloride and nitrite ions [3]. A co-culture of two bacterium, *Enterobacter cloacae* and *Alcaligenes* sp.TK-2 degraded 4C2NP with release of chloride and ammonium ions [6]. Another bacterium, *Exiguobacterium* sp. PMA degraded 4C2NP with release of ammonium and chloride ions [1]. In the case of *Pseudomonas* sp. JHN, we have not detected chloride, ammonium and nitrite ions during the transformation of 4C2NP. This data suggests that the transformation product should have chloro and nitrogen atoms.

Apart of biodegradation of 4C2NP, several reports have been published with degradation of 4C2NP by physicochemical methods [11–13]. Mehrizad *et al*. [11] used single-walled and multi-walled carbon nanotubes for removal of 4C2NP from aqueous solutions through absorption. Gharbani *et al*. [12] studied the 4C2NP degradation in the pharmaceutical industrial wastewater by ozonation and identified chlorophenol as degradation product. Adami and Fakhri [13] studied the removal of 4C2NP from aqueous solutions using zero valent iron nanoparticles (nZVI) and Pd-doped zero valent iron nanoparticles (Pd-nZVI). These physicochemical methods are not as effective as bacterial degradation because they are not cost effective [2].

**Figure 6:**
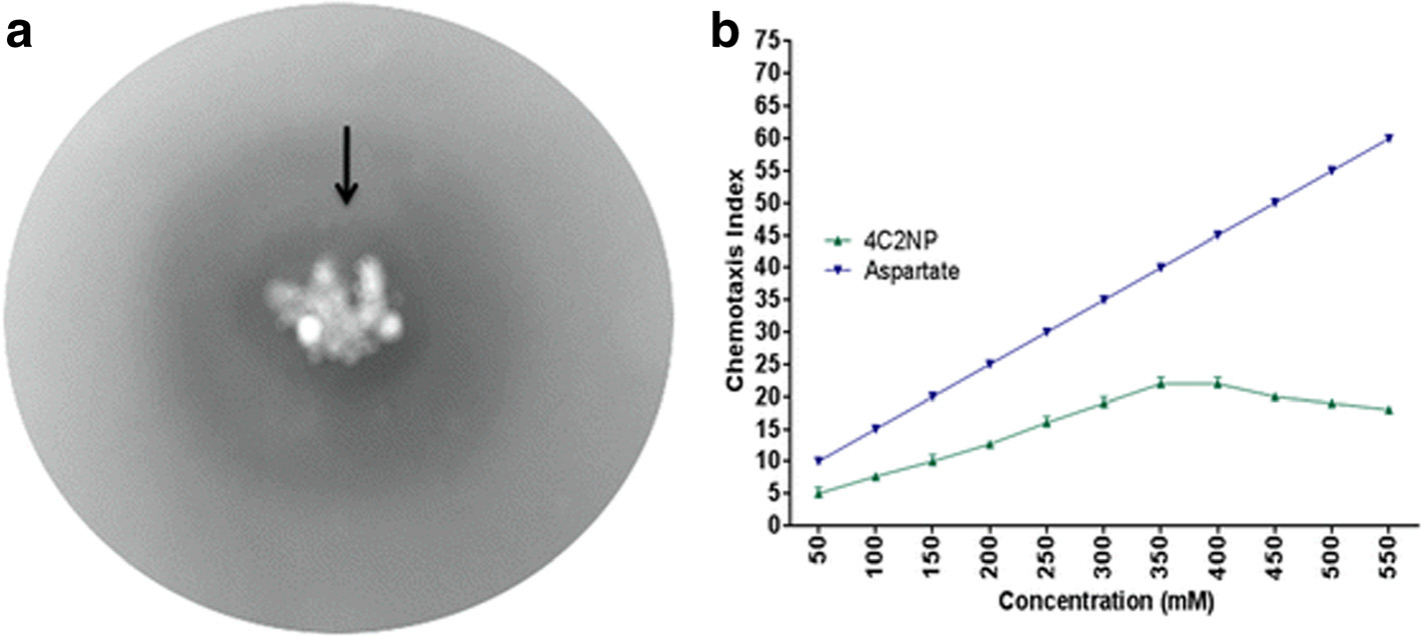
**Chemotaxis towards 4C2NP. (a)** Drop plate assay showing the bacterial ring around the crystals of 4C2NP. **(b)** Capillary assay showing the different chemotaxis index value toward different concentrations of 4C2NP. Aspartate was used as a positive control.

We have also monitored the chemotactic behavior of *Pseudomonas* sp. JHN toward 4C2NP using drop plate assay and capillary assay. Drop plate assay showed the formation of the bacterial ring around the crystals of 4C2NP after the incubation of 6 h (Figure 6a). In capillary assay, it was observed that *Pseudomonas* sp. JHN was chemotactic toward 4C2NP at an optimum concentration of 350 μM with a chemotaxis index of 22. Figure 6b show that the chemotaxis index value gradually increased with increased the concentration of 4C2NP up to optimal concentration (350 μM). After reaching the optimum concentration, there is no significant change in chemotaxis index value for 4C2NP. To the best of our knowledge, this is the first report of bacterial chemotaxis toward 4C2NP.

*Pseudomonas* sp. JHN also showed chemotaxis toward 4-chloro-3-nitrophenol and mineralized it via formation of chlororesorcinol [7]. *Pseudomonas* sp. JHN showed chemotaxis toward those compounds which it can metabolize or cometabolize. Strain JHN did not show chemotaxis those compounds which are not degraded or transformed by strain JHN. This kind of chemotaxis is known as metabolism-dependent chemotaxis [14]. Another type of chemotaxis is metabolism-independent chemotaxis which is independent of metabolism of chemo-attractants [14]. Both metabolism-independent and metabolism-dependent type chemotaxis have been studied in bacteria [14]. *Pseudomonas* sp. strain WBC-3 and *P. putida* PRS2000 exhibited metabolism-independent chemotaxis towards various aromatic compounds [15–18]. Another bacterium, *Burkholderia* sp. SJ98 [19] has been characterized for its metabolism-dependent chemotactic behavior toward various nitroaromatic compounds. Pandey *et al*. [10] reported that strain SJ98 also exhibited chemotactic response toward chloronitrophenols including 2-chloro-4-nitrophenol and 2-chloro-3-nitrophenol [10]. However, strain SJ98 neither utilizes 4C2NP nor shows chemotactic behavior toward 4C2NP [10]. We have shown chemotactic behavior of *Pseudomonas* sp. JHN toward 4C2NP in our current report. It has been proved that chemotaxis enhanced bioavailability of chemicals to the bacteria that is helpful for bioremediation processes [20].

## Conclusion

*Pseudomonas* sp. JHN transformed 4C2NP into 5-chloro-2-methylbenzoxazole and showed chemotaxis toward 4C2NP. To the best of our knowledge, this is the first report of bacterial chemotaxis toward 4C2NP.
